# The impact of Abdominal Wall Hernia (AWH) on patients’ social and sexual relationships: a Qualitative Analysis

**DOI:** 10.1007/s10029-025-03414-8

**Published:** 2025-07-16

**Authors:** Olivia Smith, Asim Abbas, Mark Mierzwinski, Veronica Oliver-Jenkins, Praminthra Chitsabesan, Srinivas Chintapatla

**Affiliations:** 1 York Abdominal Wall Unit, Department of General Surgery, York & Scarborough Teaching Hospitals NHS Foundation Trust, Wigginton Road, York, YO31 8HE UK; 2 School of Science, Technology and Health, York St. John University, Lord Mayor’s Walk, York, YO31 7EX UK; 3 Department of Psychological Medicine, York Teaching Hospital, Wigginton Road, York, YO31 8HE UK

**Keywords:** Patient reported quality of life, Sexual relationships, Social relationships, Abdominal wall hernia

## Abstract

**Background:**

Abdominal Wall Hernia (AWH) impacts interpersonal relationships, which are vital to human wellbeing [1, 2], however social and sexual dimensions of AWH remain underexplored. This study investigates the impact of AWH on social disconnection and sexual intimacy challenges, and how this could be viably assessed in health related quality of life (HRQoL) assessment tools.

**Methods:**

A qualitative approach using Interpretative Phenomenological Analysis (IPA) was employed [3]. Fifteen participants (8 men and 7 women, aged 36–85 years) were purposively sampled. Data collected via semi-structured interviews was analysed iteratively until no new themes emerged [4].

**Results:**

AWH significantly impacted participants' interpersonal relationships, mainly through ‘difficulties in connecting socially’ and ‘changes in sexual relationships’. Participants' social withdrawal was driven by physical limitations, altered self-perception, and stigma, leading to loneliness and reduced engagement. Changes in sexual relationships were shaped by pain, body image concerns, and perceived sexual pressure, often diminishing physical intimacy. Despite these challenges, emotional intimacy frequently persisted, largely due to empathetic and supportive partners. Postoperative improvements were highlighted as pivotal in restoring body confidence and intimacy.

**Conclusions:**

This study illustrates the profound social and sexual disruptions AWH causes. These effects should be included in holistic and patient centred care, and incorporated into HRQoL assessment tools. Preoperative counselling should include issues of social connection and intimacy, whilst postoperative care should integrate structured support networks, psychoeducational interventions, and psychosexual counselling. Future research should pilot, revise and test the effectiveness of such measures.

**Supplementary Information:**

The online version contains supplementary material available at 10.1007/s10029-025-03414-8.

## Background

Interpersonal relationships are vital to human wellbeing because they are deeply tied to social, psychological and emotional health [5, 6]. Whilst Abdominal wall hernia (AWH) is a chronic, visible and physically debilitating condition, it can also profoundly impact patients’ interpersonal relationships [7]. Impacts include social isolation, altered family dynamics, and disruptions to intimate partnerships. These may contribute to withdrawal from social and relational activities, disrupting and negatively affecting patients’ quality of life.

Despite this, Health Related Quality of Life (HRQoL) assessments in AWH often focus on functional outcomes, neglecting psychosocial and relational dimensions [8]. This gap may be due to a lack of patient involvement in developing these tools, thus failing to appreciate AWH impact on interpersonal connections. Similarly, the primary aim of treatments such as Complex Abdominal Wall Reconstruction (CAWR) is to restore abdominal function and prevent complications. Therefore, the impacts such treatments have on patients’ interpersonal relationships remain underexplored.

By adopting a phenomenological approach, this study delves into the impact patients’ AWH had on their social and sexual relationships.

## Methods

### Study design

The study applied phenomenology, a research methodology frequently used to qualitatively explore human issues and emotionally laden subjects [2, 9, 10]. Deep insights into how AWH affected patients’ interpersonal relationships were gained through 15 semi-structured interviews. Patients’ narratives were analysed using Interpretative Phenomenological Analysis (IPA) [11]. Data were triangulated by two abdominal wall hernia surgeons and a qualitative researcher to ensure consistency.

### Ethics

Ethics approval was received from the Hull York Medical School (HYMS), Integrated Research Approval System (IRAS) and Health Research Authority (HRA) of the United Kingdom (Research Ethics Committee (REC) reference: 19/SC/0565). Written and verbal informed consent was gained from all participants. We confirm adherence to the tenants of the Declaration of Helsinki and the Consolidated criteria for REporting Qualitative (COREQ) guidelines. A completed COREQ checklist is included as Supplementary File 1 to provide transparency regarding the design, conduct, and reporting of this study. All participant names used in this manuscript are pseudonyms, assigned to protect the identity and confidentiality of individuals in accordance with ethical approval and informed consent protocols. Pseudonyms were selected, rather than numerical identifiers, to preserve the human richness of participants’ narratives and to reflect the personal nature of their experiences.

### Recruitment

Participants were recruited from our AWH clinics using purposive sampling, a qualitative research technique designed to identify “information rich cases” [4]. This sampling approach ensured pre- and post-operative patients with diverse experiences across all Ventral Hernia Working Group (VHWG) grades 1–4 were included.

This approach meant that recruited individuals often had comorbidities such as prior wound infections, stoma, intestinal fistula, diabetes, smoking history, and obesity [12]. Furthermore, participants with a history of colorectal cancer, inflammatory bowel disease, and varying socioeconomic and employment statuses were also recruited. While open to individuals of any ethnicity, the predominantly white British demographic of the region was reflected in the sample. Due to the complexity and heterogeneity of hernias in this cohort (e.g. parastomal or recurrent incisional hernias), EHS classification was not uniformly applicable and has therefore not been used as a classification framework in this study. Supplementary File 2 provides participants’ demographics, including CT-documented hernia size and comorbidities, offering additional context to narratives presented below.

Participants received a letter of invitation accompanied by an information sheet detailing the study’s aims and interview process. Collectively, this recruitment approach enabled varied narratives to be captured, including the impact AWH had on patients’ interpersonal relationships. A more detailed overview of the recruitment methodology is available in supplementary project publications [1, 7].

### Research method

To gain rich and narrative style responses, semi-structured interviewing techniques were adopted [3, 13]. The interview process (Supplementary File 4) was supported by a schedule and topic guide, developed by qualitative researchers, two consultant plastic surgeons and two consultant gastrointestinal surgeons who operate within the Abdominal Wall Unit. The interview guide contained four specific questions relating to social and sexual relationships (Supplementary File 3, topic f).

### Data collection

Fifteen interviews were conducted by author OS, a Research Fellow, and reviewed by MM. Three interviews were face to face, whilst the rest were conducted by telephone due to the Covid-19 pandemic. Interviews lasted between 45 and 90 min. Trustworthiness was ensured by asking open questions, clarity checks, and by using prompts and probes (Supplementary File 3, topic f). Audio-recorded interviews were transcribed verbatim by OS and a medical secretary independent of the research team. Pseudonyms were used to ensure anonymity and confidentiality.

### Data analysis

Data were analysed iteratively until thematic saturation. Thematic saturation was determined when no new codes or themes emerged during the final three interviews, indicating that the data had become repetitive and comprehensive. The research team reviewed each transcript iteratively, and saturation was discussed collectively during regular analysis meetings to ensure consensus and analytic completeness. Transcripts were analysed using IPA within NVivo v12 (https://www.qsinternational.com/nvivo/home). Similar participant views were grouped into superordinate and subordinate themes concerning interpersonal relationships. Emergent themes were triangulated with an independent qualitative researcher (MM) as well as gastrointestinal surgeons (SC, PC) who specialise in CAWR thereby allowing triangulation of the findings and increasing plausibility of results. This process helped ensure that the interpretations reflected the participants’ narratives rather than the biases or assumptions of a single researcher, thus reducing the risk of over-interpreting or inadvertently leading responses.

## Results

**Fig. 1:**
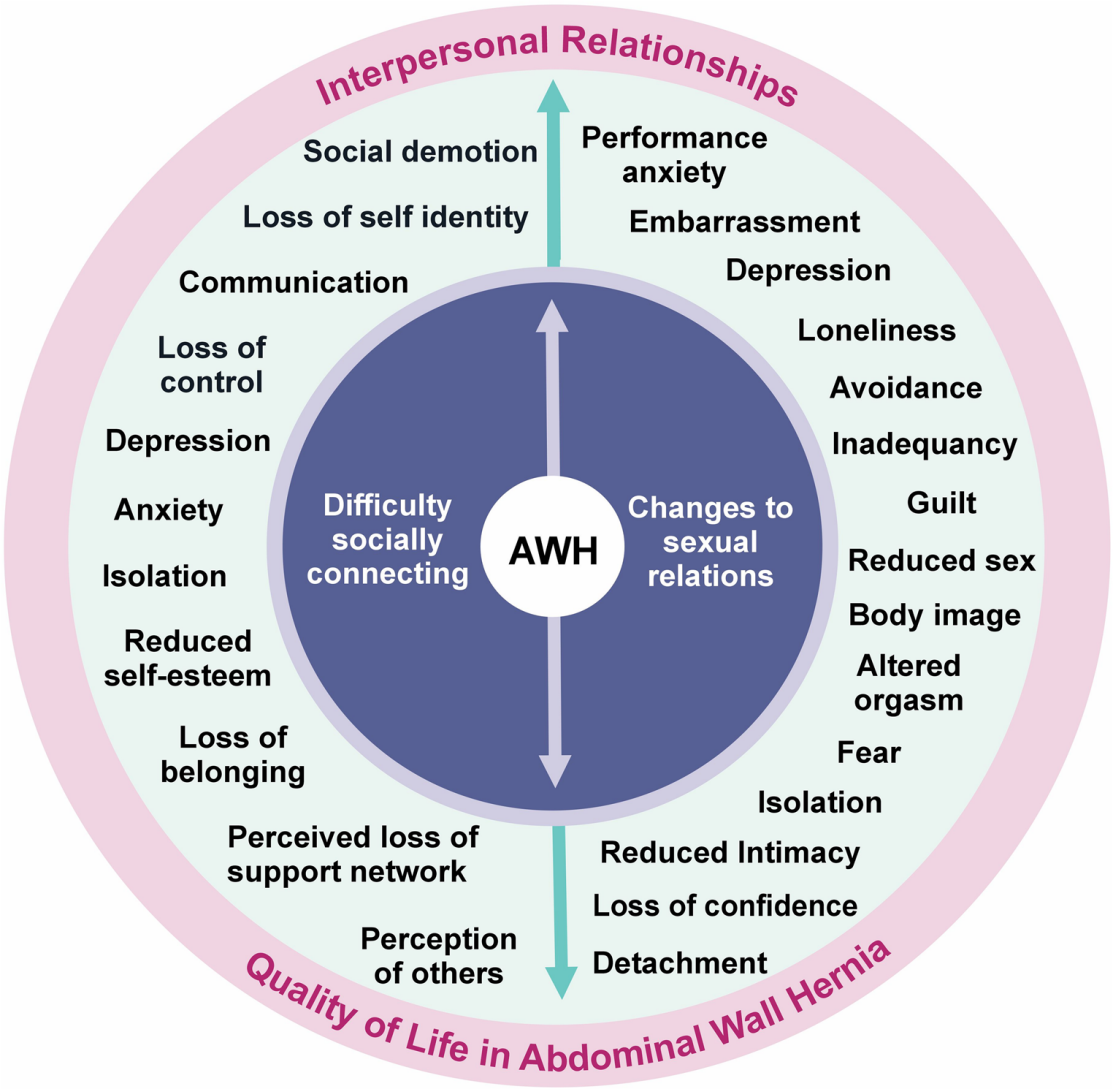
Interpersonal relationship themes relating to patients’ quality of life

Fifteen participants took part in this study (8 men and 7 women) with an age range of 36–85 years (median = 65 years; interquartile range = 30 (45–75) years). Supplementary File 2 provides a summary of participant characteristics. Supplementary File 5 provides participants’ biographies, offering additional context to narratives presented below. When analysing the impact AWH had on patients’ interpersonal relationship, two primary superordinate themes emerged: Difficulties in Connecting Socially and Changes in Sexual Relationships, which had 11 and 15 subordinate themes respectively [7] – see Fig. 1.

### Theme one: difficulties in connecting socially

Many participants described a profound sense of social disconnection, attributed to the combined impact of physical, psychological and emotional burdens. Patients articulated how their AWH’s bio-psycho-emotional-social impact detrimentally affected their self-perception, levels of anxiety, and perceived stigma. These effects manifested as withdrawal from social settings, altering family dynamics, and fostering avoidance behaviours rooted in a heightened self-consciousness.

### Social withdrawal and isolation

12 patients expressed social withdrawal and isolation from their social lives. Withdrawal was driven by various factors, including physical limitations, negative self-perception, and the fear of judgment or misunderstanding.

*I just never felt happy going out into a big social situation without feeling massively self-conscious about the way I looked (Ophelia*,* a 44-year-old mother of two)*.

*It affected my social life as well…I used to enjoy having a drink and socializing*,* but I don’t bother anymore now. (George*,* a 45-year-old tradesman)*

*I’ve always*,* always tucked things in but since the operation really*,* it shouldn’t matter in some ways*,* but it does…I’ve got to have looser clothes. I mean*,* if I can’t wear the belt again*,* my tummy looks*,* well*,* to me it looks awful. (Joan*,* a 75-year-old retiree)*

Collectively, these quotes illustrate patients’ appearance related heightened self-consciousness, largely informed by a growing fear of being (more) judged. Avoidance behaviours, disengagement from social interactions, and changes in their relationship dynamics compounded participants’ feelings of loneliness and disconnection.

More specifically, social withdrawal brought changes in how participants engaged with family, hobbies and occupations, and this was further disrupted by the manner in which AWH affected some participants’ identity and role within their family or professional life.

*I’ve had to learn how to ask for help*,* and it’s quite a strange sort of thing to have to start doing*,* you know*,* to reprogram your mind. (Ian*,* a 58-year-old grandfather)*

*I’ve always been a cyclist and I had a tandem which I used to go out with my grandson on*,* because of his disabilities…When I came out of hospital I got rid of the tandem and the butcher’s bike. (Frank*,* a 75-year-old grandfather)*

*I started to seriously contemplate well maybe I shouldn’t be a PE teacher*,* maybe I should start thinking about teaching another subject. (Lisa*,* a 39-year-old physical education teacher)*

It became clear that participants’ diminishing physical contributions to family/work life and growing dependency on others with an associated diminishing self-worth caused frustration, sadness and feelings of helplessness.

Some participants adapted their behaviours in response to living with AWH. For instance, Frank began using a reclining tricycle to accommodate his physical limitations while Joan, a 75-year-old retiree, shifted from attending plays and dancing to actively participating in hernia and stoma support groups. These groups offered her an emotional outlet, where she could express distress about her self-perceived “ugly” abdomen, and helped alleviate some of the social isolation she felt from no longer engaging in her former activities. In contrast, individuals like Eric - a 78-year-old retired athlete - withdrew from previous hobbies such as gardening. These examples illustrate that although 12 participants reported experiencing social withdrawal, those who had previously led active or extroverted lifestyles were particularly affected. Overall, the findings suggest that as participants adjusted to life with AWH, the resulting physical and aesthetic changes often created barriers to sustaining social relationships - leading many into a pattern of avoidance and isolation.

### Emotional reactions to family support and coping strategies

Reflecting upon their growing dependency on others, participants frequently cited how their AHW had strengthened bonds with family members and friends. Narratives were laden with emotional phrases such as “*she is my rock*” (George, a 45-year-old tradesman), whilst Betty (a 63-year-old former caregiver) cited “*You go on struggling until someone says*,* come on*,* stop struggling*”. In Betty’s case, her children’s proactive assistance, such as taking time off work to help her and her husband, became a critical support system. Whilst spousal support was significant in physically and emotionally managing living with AWH, it could foster feelings of guilt. Wishing to simply “*get on with his life*”, Ian (a 58-year-old grandfather) expressed her frustration at becoming “*burdensome*” to his children at a time when they welcomed his first grandchild. However, he found inspiration in experiencing the cycle of life, vowing to “*never to waste another day*”. Feelings of guilt emerged as a recurring theme among participants, reflecting tensions between independence and reliance on others for support. This process required changing and/or new perspectives and evidenced the emotional complexity of navigating dependency, where frustration and gratitude often coexisted.

Participants without close family support developed various coping mechanisms to navigate their AWH social realities. While some found engagement in support groups therapeutic (i.e. Joan), others adjusted their daily social interactions to manage everyday interactions. Many participants avoided public spaces or activities to mitigate discomfort or perceived stigma.

*I have to think of routes where there are other people about…it’s just constantly at the back of my head…am I in a position where I could be got somewhere to get to A&E. (Agnes*,* a 65-year-old retiree)*

*If she wanted a cuddle (daughter)*,* I would sit on the sofa without picking her up…So I just adapted. But yeah*,* it wasn’t very nice. (Marge*,* a 36-year-old mother of three)*

Illustrating how participants coped with the intersecting challenges of pain, embarrassment, and self-consciousness, such reprioritisations and modifications of daily activities reflected a dual sense of loss and accommodation. Therefore, whilst often pragmatic, participants’ narratives revealed the psychological toll of making such adaptations, which was often described as providing only temporary or partial relief.

Faced with such daily adversity, humour was a coping mechanism for some participants, being primarily used as a linguistic shield and to bridge social awkwardness. For instance, when citing physical restrictions, Ian smirked and said, “*I would*,* of course*,* use the correct lifting [laughs]*,* like we all do”*. This lighthearted approach helped deflect focus from his limitations while maintaining a sense of normalcy in social exchanges. Furthermore, when responding to mistaken assumptions about her appearance, Ophelia (44-year-old mother of two) cited how, “*I was embarrassed*,* and I’d correct them in a sort of joking*,* laughing it off kind of way*”. Whilst such humour temporarily appeased social awkwardness, Ophelia disclosed how, “*I’d come away from that situation thinking*,* God that’s made me feel really crappy*”. These accounts demonstrate how, while providing momentary relief, humour often masked participants’ feelings of shame and frustration. The complex interplay between humour as both a coping strategy and a reflection of deeper insecurities underscores its role in navigating the social disconnection experienced by those with AWH.

### Theme two: changes in sexual relationships

Sexual relationships are an essential component of quality of life, and this study highlights the profound impact of AWH on intimacy and sexual dynamics. When asked if their AWH affected their relationships with their spouse/partner and sexual intercourse, participants described adverse effects. Struggles to engage in sexual activities led to frustration, vulnerability, and challenges in maintaining emotional and physical intimacy.

### Physical and emotional barriers to sexual activity

Physical discomfort and pain were the most cited reasons for disrupted sexual activity among participants.

*The sex life has gone down and everything*,* because of that (the hernia). It hurts me doing anything like that. Everything’s sort of had a knock on effect for me (George*,* 45-year-old tradesman)*.

*The sex isn’t what it was because it’s hurting too much but…we’re as much in love now as we ever were. (Charlotte*,* a 68-year-old retiree)*

*The intimacy has gone as the wife keeps reminding me…if I could remember what it [intercourse] is. (David*,* 61-year-old grandfather)*

These examples demonstrate how physically informed disruptions to sexual activity were frustrating and impacted intimacy levels to differing degrees. Many participants also cited emotionally laden reasons for withdrawing from sexual activity. The main reason was due to the perceived unsightliness of their AWH which had instigated insecurities when naked in front of their partners.

*You tend not to look at yourself anymore because it really is quite a horrible looking thing and I avoid mirrors and my wife’s not allowed to look at me anymore. (Eric*,* 78-year-old retired athlete)*

*The way I feel about being naked I suppose has had an impact on our sexual relationship. (Lisa*,* 39-year-old PE teacher*,* mother of two)*

Kevin (74-year-old retiree) who was interviewed with his partner present, shared how his partner would have to be blindfolded to help him dress, and that he found this embarrassing. Collectively, narratives such as these revealed participants’ heightened feelings of vulnerability, anxiety and embarrassment surrounding their sexual attractiveness. Fears of how their partners perceived them compounded their own disgust and/or dismay at the aesthetic of their AWH, which could lead to feelings of inadequacy and guilt from unmet expectations within a marital relationship. Whilst most participants expressed supportive and understanding partners, i.e. “*My partner was always very sensitive*” (Ophelia, 44-year-old mother of two), they still reflected upon relational tensions and degrees of social disconnection that AWH had caused them.

Finally, those interviewed post-operatively revealed changes in their sexual relationships. Describing her operation as a turning point, Lisa (39-year-old PE teacher and mother of two) stated, “*The feeling (having sex) is definitely different now. I don’t know whether that’s because of the hernia but…there is a marked improvement after the operation*”. In this respect, there were little negative effects cited concerning post-surgery. Indeed, the prevailing narrative was one whereby physical recovery aided the ability to restore levels of sexual intimacy, whilst surgery also contributed to a psychological restoring of body confidence.

## Discussion

The results of this study, visually summarised in Fig. 1, and presented above demonstrate how AWH caused patients ‘difficulty in socially connecting’. Not merely due to physical issues, social disconnection was deeply rooted in psychological, emotional and social factors [14]. These factors often manifested feelings of social demotion, where altered physical appearances and functions disrupted participants’ roles within their family, work, and social settings. Feelings of social demotion were reflected by how individuals exhibited behaviours of avoidance, shame, or a perceived threat to social status [15]. Disrupted roles and negative emotions often led to social withdrawal and social isolation.

Coping strategies often temporarily masked and/or managed feelings of inadequacy, embarrassment or frustration. Whilst some participants’ use of self-defeating humour deflected attention away from or made light of their condition, it also served to reinforce inadequacy and stigma [16]. The few participants who accessed structured support groups reported favourably on their ability to foster resilience, offer confidentiality and provide a platform to share frustrations [17]. The most reliable, consistent and effective coping mechanism was close family physical and emotional support [18]. However, such offerings and degrees of dependency often induced shame in a society that encourages and values individuation and self-sufficiency [19].

AWH caused ‘changes in [patients’] sexual relationship’, adversely affecting patients’ physical, psychological and emotional ability to engage in sexual activities [14]. Chronic pain led to decreased frequency of sexual activity and levels of satisfaction [20]. Such decreases contributed to many participants’ heightened negative body image, particularly concerning being naked [21]. Compounding feelings of inadequacy and shame, such decreases fostered high levels of anxiety and low levels of self-esteem. However, some participants described a “sexual intimacy paradox”, where couples in communicative relationships maintain emotional closeness even as physical intimacy diminishes [22]. Physical intimacy improved post-CAWH surgery/recovery due to physical and psychological improvements.

### Study limitations

This study offers rich patient narratives, which produces subjective knowledge desired for attaining patient centred quality of life experiences. To ensure credibility and trustworthiness, rigorous methodological steps were employed, such as purposive sampling, triangulation, and adherence to analysing until no new themes emerged. Because of the COVID-19 pandemic, much data was collected via telephone interviews. This method fails to capture non-verbal cues such as body language and unspoken emotions during sensitive discussions concerning social relationships and intimacy. To mitigate this limitation, the interviewer (OS) used reflective listening techniques, verbal affirmations, and careful probing to encourage depth and emotional nuance in participant responses. Field notes supported the detailing of subtle cues that may have been at risk of overlooking. Open ended questions, follow up prompts, and frequent participant-led clarification were employed to ensure that subtle meanings and emotional content were captured as fully as possible through verbal communication alone.

The cross-sectional nature of this study meant that participants’ experiences were captured at a single time point. This approach does not capture changes in social and sexual relationships throughout the AWH journey, particularly in response to preoperative counselling or postoperative recovery. Finally, the findings are reflective of the Caucasian (97%) and middle class (73%) sample. Whilst representative of the population of North Yorkshire, United Kingdom, this homogeneity limits the generalisability of findings to more ethnically and socioeconomically diverse populations.

The role of the interviewer (OS) also warrants consideration. OS is a cispresenting, Caucasian, young, female doctor with surgical and research training, as well as operative experience in CAWH. She had no prior relationship with them before the interviews. Nonetheless, OS engaged in training on cognitive interviewing prior to interviews, and active reflexivity throughout the study, recognising that her demographic and professional background may have shaped how participants related to her, and potentially influenced how the data were interpreted. This reflexive stance was supported by regular analytic discussions with the broader research team, including those outside of surgical practice, to reduce bias and support balanced interpretation.

### Development suggestions

Our findings highlight a significant gap in current HRQoL assessment tools for patients with AWH, particularly regarding the psychosocial domains of social connection and sexual relationships. In our companion study - a critical COSMIN-informed scoping review of CAWH-specific HRQoL tools (in press) - we found that while existing instruments offer clinical utility, none fully capture the interpersonal and psychosexual challenges identified in this study. As an example, the HerQLes tool includes assessment of sexual dysfunction, but coverage is limited to a single, narrowly framed item. No currently available tool comprehensively addresses the impact of AWH on broader sexual intimacy or social withdrawal [8], which were recurring and deeply felt themes in our interviews. This reflects a wider issue in tool development: a lack of patient involvement in content generation and insufficient attention to relational quality of life outcomes. Future development or refinement of HRQoL tools should therefore prioritise (a) codevelopment in partnership with patients to capture nuanced relational impacts; (b) inclusion of validated items addressing sexual intimacy, body image, and social identity; and (c) alignment with COSMIN standards to ensure both psychometric robustness and clinical relevance. Incorporating these domains would enable more holistic assessment of treatment outcomes and better guide supportive interventions.

Given how AWH detrimentally affected patients’ quality of life - particularly through social disconnection and disrupted sexual intimacy - holistic care must encompass physical, psychosocial and psychosexual dimensions. Preoperative counselling offers a valuable opportunity to acknowledge and validate patients’ emotional challenges, such as feelings of guilt and fears of dependency, whilst promoting a balanced approach to maintaining independence whilst accepting necessary support. It should also prepare patients and their partners for potential changes in intimacy. Addressing pain effectively is essential not only for physical recovery, but also vital for restoring sexual function and emotional intimacy. Postoperative care should integrate tailored interventions to rebuild confidence and connection by drawing on approaches used in other contexts, such as psychosexual interventions for post-mastectomy patients, incorporating body image therapies and communication training could provide critical support [23, 24]. Similarly, lessons from stoma patients emphasize the importance of addressing anticipated sexual challenges early, equipping patients with coping strategies to navigate these sensitive issues [25].

**Fig. 2:**
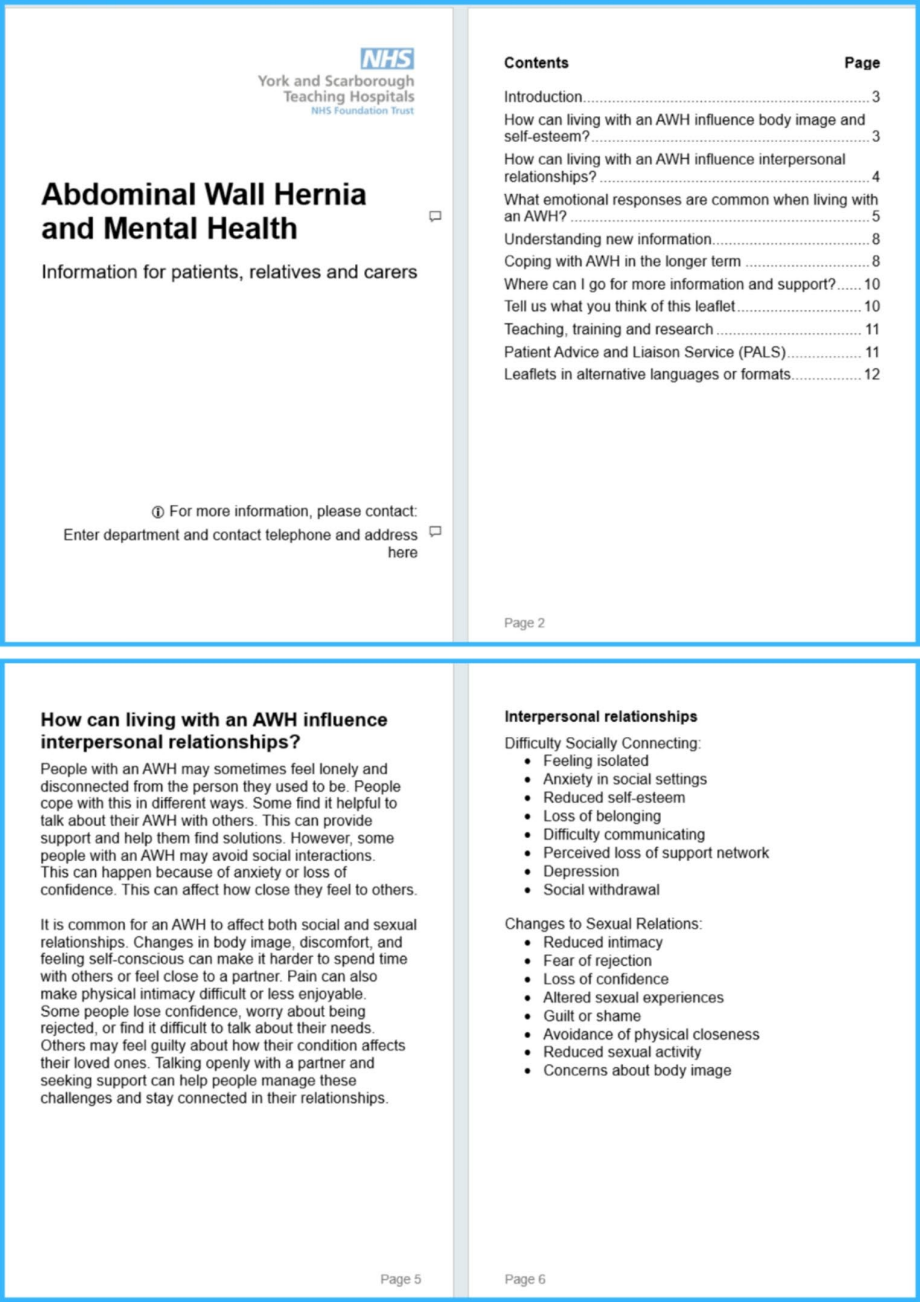
Codeveloped Patient Information Leaflet (PIL) on ‘AWH and Mental Health’

In response to these unmet needs, we have developed a patient information leaflet on “AWH and Mental Health” in collaboration with patient representatives and a clinical psychologist. The leaflet addresses issues raised in this study - including sexual and social relationships, physical intimacy, and difficulties in socially connecting - and has been written in Plain English, with a Flesch Reading Ease score of 63.9, to ensure accessibility. Drawing directly from the narratives of individuals living with AWH, the leaflet explains how the condition can affect confidence, self-esteem, and communication, especially in relation to close relationships. It encourages open dialogue, validation of patients’ emotional responses, and recognition that self-blame is common but misplaced. This resource was designed as part of a wider programme of work identifying all domains of quality of life impacted by AWH [7], focussing on mental health [26], body image [1], and interpersonal relationships, and aims to offer a more holistic and psychologically informed approach to patient support. The leaflet brings together these interconnected domains to provide practical, empathetic guidance for patients and their loved ones. A section of the leaflet is in Fig. 2.

**Fig. 3:**
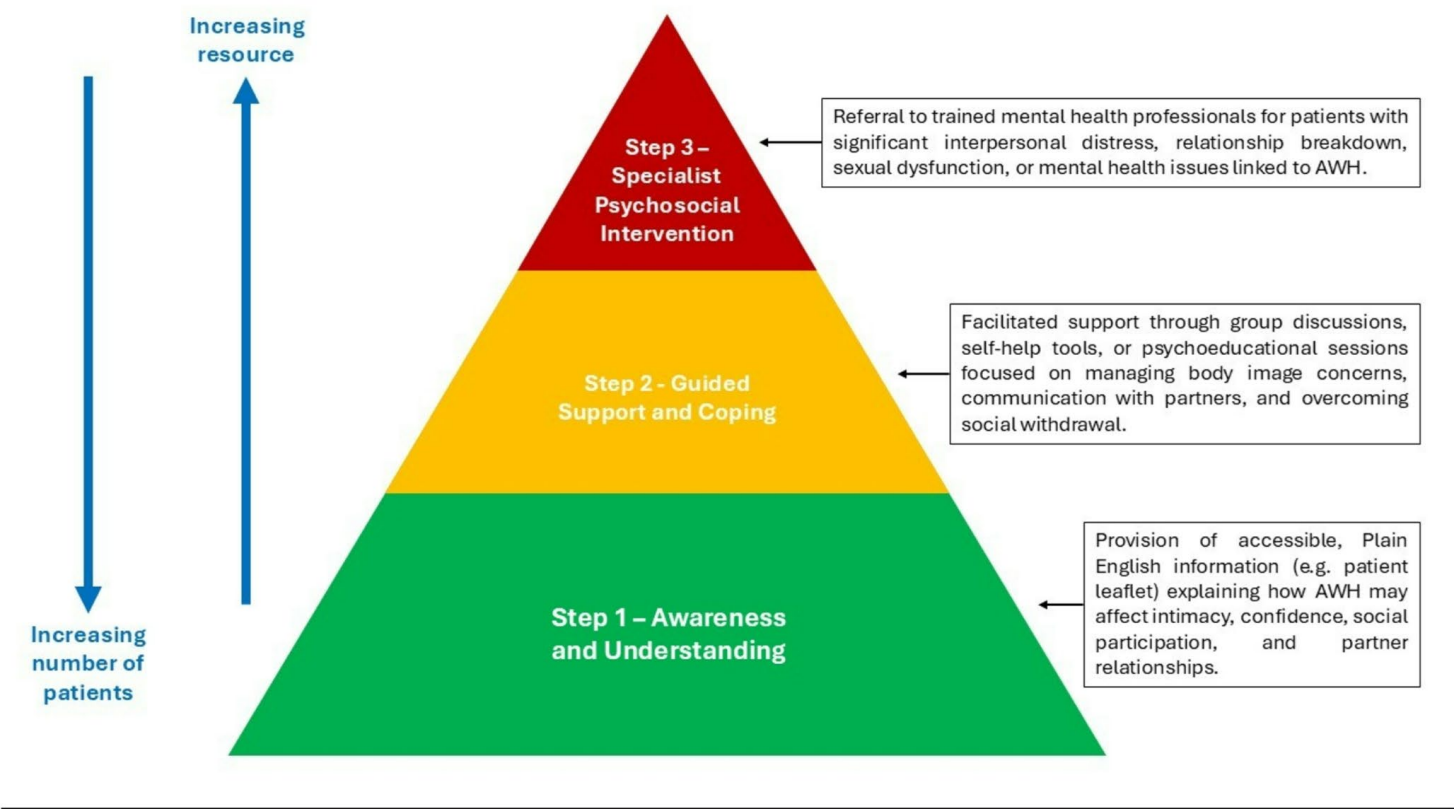
A stepped care approach to sexual issues in patients with AWH

The leaflet is one example of Step 1 “Awareness and Understanding” within a stepped care model, which recognises that not all patients require the same level of psychological intervention. Step 2 “Guided Support and Coping” might involve psychoeducational support such as facilitated group discussions or guided self-help programmes focused on sexual dysfunction, social confidence, and relational challenges, tailored to those expressing mild to moderate distress. Step 3 “Specialist Psychosocial Intervention” could involve referral to a trained mental health professional for patients experiencing more significant psychological disruption, offering specialist assessment and therapeutic intervention. Surgical repair is not explicitly represented here, as the model is designed to offer support before and after any surgical intervention, or for patients who are not surgical candidates. Therefore, all three steps can apply regardless of surgical status, ensuring a holistic patient-centered approach. Figure 3 is a visual representation of this stepped care approach.

## Conclusions

This study is the first to qualitatively examine how AWH detrimentally affects patients’ social and sexual relationships, impacting their quality of life. Narratives concerning social disconnections and loss of sexual intimacy evidenced the interplay between physical, psychological, and relational factors. If HRQoL assessment tools are going to be patient centred and informed, then future measures must account for social and sexual wellbeing to provide a more comprehensive understanding of patient needs. Furthermore, preoperative consultations should proactively explore challenges patients face and tailor interventions to address these needs. Postoperative care should integrate structured support networks, psychoeducational programs, and psychosexual counselling. Drawing from insights in related fields, step-wise interventions such as peer and group support programs, relationship counselling, self-esteem and body image therapy, could offer support in mitigating the psychosocial impacts of AWH.

By advancing our understanding of the multidimensional consequences of AWH, this study highlights the importance of integrated care approaches that prioritize not only physical recovery but also emotional and relational wellbeing. However, further research should explore changes in social and sexual relationships throughout patients’ AWH journey. Whilst not feasible in this study, this process could include validating patients’ experiences by gaining the pellucid viewpoints of partners, family members, and caregivers. Finally, as cultural and socioeconomic factors may influence patients’ perceptions and experiences of social disconnection and changes to sexual relations, future studies should seek a broader demographic as a sample.

## Electronic supplementary material

Below is the link to the electronic supplementary material.

Supplementary Material 1

Supplementary Material 2

Supplementary Material 3

Supplementary Material 4

Supplementary Material 5
