# Supplementary figures and images for: The impact of Abdominal Wall Hernia (AWH) on patients’ social and sexual relationships: a Qualitative Analysis
Source: Hernia. 2025 Jul 16;29(1):234. doi: 10.1007/s10029-025-03414-8 (PMC12267305; doi:10.1007/s10029-025-03414-8)

**Supplementary File 4:** interview schedule


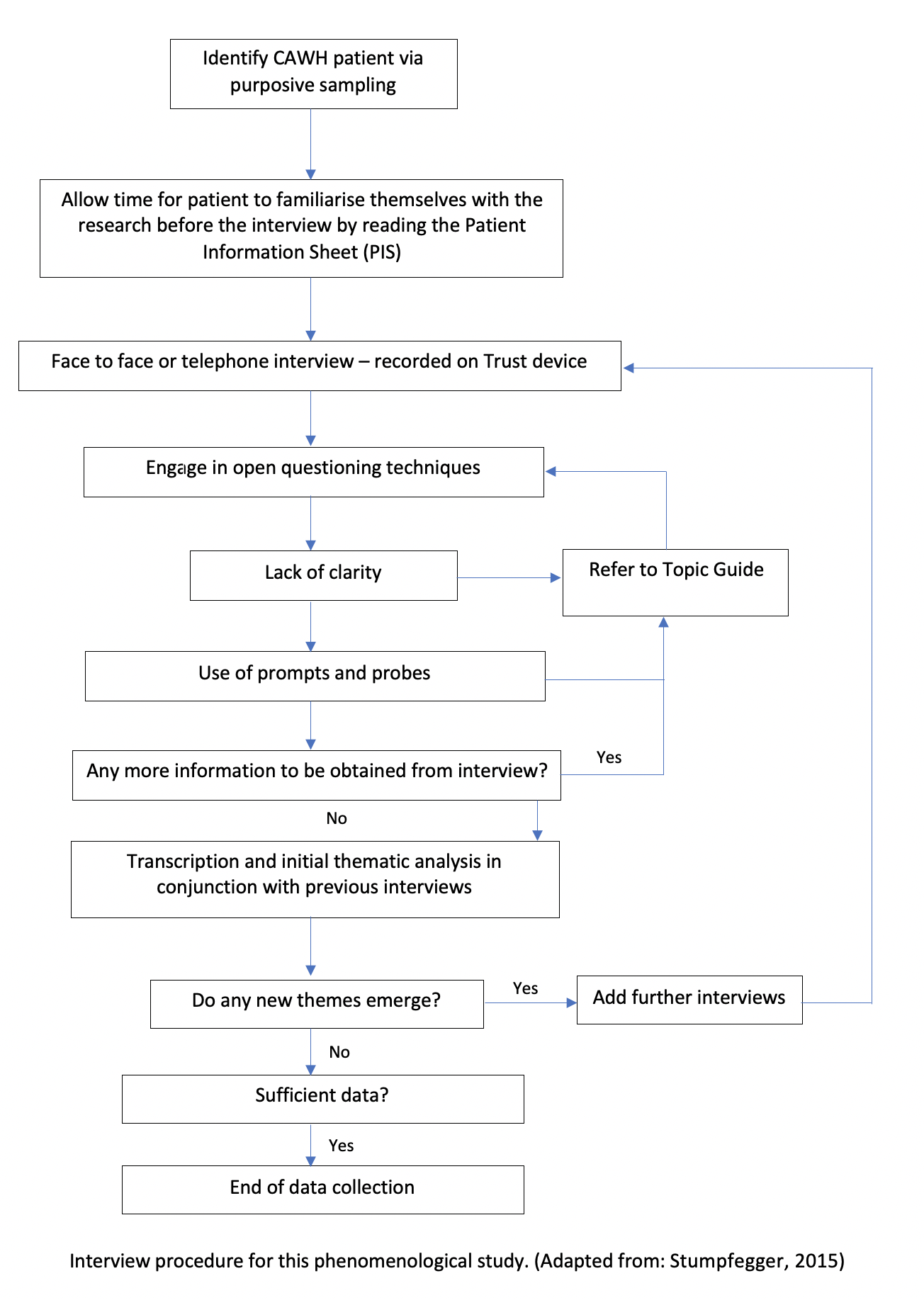

Supplement: Supplementary file 4 — Supplementary Material 4 [file 10029_2025_3414_MOESM4_ESM.docx]
